# Editorial: Difficult and severe asthma in children, volume II

**DOI:** 10.3389/fped.2023.1158309

**Published:** 2023-03-16

**Authors:** Nicola Ullmann, Andrew Bush, Giorgio Piacentini, Francesca Santamaria, Renato Cutrera

**Affiliations:** ^1^ Pneumology and Cystic Fibrosis Unit, Academic Department of Pediatrics, Bambino Gesù Children’s Hospital, Rome, Italy; ^2^ Professor of Paediatrics, National Heart and Lung Institute, Imperial College London, London, United Kingdom; ^3^ Professor of Paediatrics and Paediatric Respirology, National Heart and Lung Institute, London, United Kingdom; ^4^ Consultant Paediatric Chest Physician, Royal Brompton Harefield NHS Foundation Trust, London, United Kingdom; ^5^ Professor of Paediatrics and Head of Paediatric Section AOUI Verona, University of Verona, Verona, Italy; ^6^ Department of Translational Medical Sciences, Pediatric Pulmonology, Federico II University, Naples, Italy

**Keywords:** severe asthma, children, treatment, mepolizumab, dupilumab, omalizumab, airway inflammation

**Editorial on the Research Topic** Difficult and severe asthma in children, volume II

This Topic covers many different aspects of childhood asthma, focusing mainly in those children suffering from severe asthma, following on from our previous book (1). As with the first volume, there is a breadth of topics, emphasizing the complexity and diversity of the manifestations of asthma. The overviews include asthma prevention during pregnancy, an exploration of the multiple comorbidities of asthma, and finally focusing on the right approach to personalizing medicine by choosing the correct drug, including the new biologic treatments, in severe asthma.

It is well known that asthma is the most prevalent chronic respiratory disease of childhood and its burden by age group is reviewed in a systematic analysis by Zhang and Zheng. Data were obtained from the Global Burden of Disease (GBD) study, which was conducted from 1990 to 2019 in 204 countries. The authors focused especially on the incidence, mortality and disability-adjusted life years (DALYs) of childhood asthma. They also update the different risk factors according to age group and region/country, emphasizing that there is geographical diversity, and asthma varies between and within countries. The obvious implication is that treatment and prevention strategies cannot be a “one size fits all”.

## Folic acid supplementation during pregnancy and risk of asthma

Asthma, defined as variable expiratory airflow restriction and recurrent respiratory symptoms, such as wheezing, shortness of breath, chest tightness, and cough, is frequently diagnosed in young children. The origins lie in complex interactions between multiple genes and environmental exposures occurring at critical periods throughout life (2). Ideally, the aim should be primary prevention of childhood asthma, or if that fail, secondary preventive strategies. Therefore, identifying risk factors and thus a high-risk population is important if early intervention is to be feasible (3). Early-life factors were associated with asthma onset throughout childhood (4) pregnancy adverse exposures are particularly important. Prenatal factors for increased risk of asthma include maternal diet and the maternal microbiome (5). Yang et al. performed a systematic review and dose-response meta-analysis to explore the relationship between maternal folic acid supplementation during pregnancy and risk of childhood asthma. They suggested that the risk of asthma in children significantly increased when maternal folic acid intake reached 581 mcg/day. However, this finding has to be set against the beneficial effects of folic acid preventing neural tube defects (6).

## Vitamin D to treat children with asthma: yes or no?

Accumulating evidence suggests that high-dose maternal vitamin D supplementation during pregnancy might reduce the risk of early life asthma/wheeze in the offspring (7) and an increasing number of studies also have suggested that vitamin D can be used to treat childhood asthma but its clinical effects are still unclear. Hao et al. performed a meta-analysis on eight randomized controlled trials and showed that vitamin D supplementation significantly increased patients' serum vitamin D levels, but it had no benefit for asthma control.

## Severe asthma and comorbidities

Although in the majority of children with asthma good outcomes are easily achieved with low-moderate dose inhaled corticosteroids if they are inhaled regularly and correctly. However, a small group with severe disease remains uncontrolled despite optimal adherence to prescribed therapy and treatment of contributory factors, including coexisting comorbidities (8, 9). In order to reduce the risk of severe exacerbations and progressive loss of lung function with the likely consequence of chronic obstructive pulmonary disease, all possible comorbidities should be assessed (10). Ronco et al. tackle this topic and nicely review all the major comorbidities that need to be taken into consideration in pediatric severe asthma.

## Severe asthma and adolescence

Adolescence is a challenging time of transition and significant differences can exist in the manifestation, exacerbating factors and management strategies for asthma (11). Hence, the adolescent patient cohort is a unique group and are the focus of the review article by Warraich and Sonnappa. The authors especially explored the main factors that may pose a challenge to the management of severe adolescent asthma whilst offering suggestions for changes in clinical practice.

## Possible treatments in severe asthma

Before escalating treatment it is important to be confident that the diagnosis is asthma and to check for social and environmental factors which are preventing a good treatment outcome. The medical evaluation should firstly include: possible associated diagnosis, assess adherence, exclude exposure to tobacco, e-cigarettes and allergens and finally, assess psychosocial factors (12).

This section on treatment is introduced by Bush with a detailed overview on how to choose the right medications for school age and preschool children with severe asthma. The main treatment approaches are presented, including also the most recently introduced medications.. In another review article Santamaria et al. summarized the pharmacological effects of the long acting muscarinic antagonist (LAMA) tiotropium bromide based on the current asthma studies at different ages, and delineating future research needs. Finally, the indications for currently available biological treatments, namely omalizumab, mepolizumab and dupilumab are presented in detail by Fenu et al. Ullmann et al. and Ferrante et al. respectively. For each biological the mechanism of action, and the major literature in relation to efficacy and safety data in children are presented.

## Summary and conclusions

This Topic offers a perspective on severe asthma in children with useful practical articles. We are grateful to all the authors have made such valuable contributions. The Editors have certainly enjoyed working on this Topic, and we hope it will be of great interest for all readers.
